# Pregnancies in women with rare diseases: Selected maternal and perinatal outcomes

**DOI:** 10.1111/aogs.70201

**Published:** 2026-04-08

**Authors:** Philipp Kosian, Kristin Niederhöfer, Elena Jost, Ulrich Gembruch, Tim Bender, Lorenz Grigull, Waltraut Maria Merz

**Affiliations:** ^1^ Department of Obstetrics and Prenatal Medicine University Hospital Bonn Bonn Germany; ^2^ Center for Rare Diseases Bonn University Hospital Bonn Bonn Germany

**Keywords:** healthcare requirements, maternity care, obstetric medicine, pregnancy, pregnancy outcome, rare disease

## Abstract

**Introduction:**

Rare diseases (RD) are characterized by chronicity and may be associated with reduced life expectancy and quality of life. Case series and reports regarding pregnancies in individuals with specific RD exist, but there is no data on the outcome of pregnancies in the overall group.

**Material and Methods:**

A retrospective analysis was conducted of all pregnancies in women with RD who were managed at our center between January 2018 and July 2022. Maternal, fetal, and obstetric parameters were recorded.

**Results:**

During the study period, 388 pregnant women with 434 RD were managed. Of these, 11.9% had more than one RD. The breakdown of conditions was as follows: 50.7% acquired diseases, 21% congenital diseases excluding malformations, 17.5% malformations, and 10.8% tumors. Disease‐specific complications occurred in 23.2% of women, and pregnancy‐specific complications in 25.1% of live births. Women with preconception stability experienced significantly fewer complications. The cesarean section rate was 50.6%. Preterm birth occurred in 15.3% of cases, and 20.4% of newborns required admission to the neonatal intensive care unit.

**Conclusions:**

Women with RD experience a high rate of disease‐specific and pregnancy complications. Preconception stability is a key factor for an uncomplicated course of pregnancy and birth.

## Graphical abstract

Pregnancies in women with rare diseases carry substantial disease‐related (23.2%) and pregnancy‐specific (25.1%) risks. Complication rates drop markedly when conditions are stable before conception.

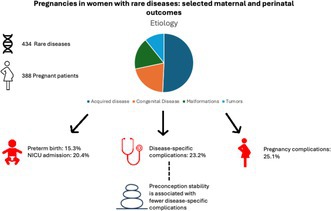

**Abbreviations**
ACOG: American College of Obstetricians and Gynecologists
BMI: body mass index
CDC: Centers for Disease Control and Prevention
CFTR: cystic fibrosis transmembrane conductance regulator
CS: cesarean section
GDM: gestational diabetes
ICP: intrahepatic cholestasis of pregnancy
ICU: intensive care unit
IUFD: intrauterine fetal death
NICU: neonatal intensive care unit
RD: rare disease(s)
SLE: systemic lupus erythematosus
SMM: severe maternal morbidity

**Key message**

Pregnancies in women with rare diseases are associated with substantial disease‐related (23.2%) and pregnancy‐specific (25.1%) complications. Preconception disease stability was associated with lower complication rates.

## 1 INTRODUCTION

In Germany, approximately 4 million people are diagnosed with a “rare disease” (RD), which in Europe is defined as a prevalence of less than 1 in 2000 individuals. In analogy to the increase in pregnancies among women with chronic medical conditions,^1^ the number of pregnancies in women with RD is on the rise.^2^ RD is characterized by chronicity and may be associated with reduced life expectancy and quality of life.^3^, ^4^ Adequate clinical care and research on the healthcare requirements is challenging due to the small cohort of affected individuals.^5^ A 2014 report of the Fraunhofer Institute for Systems and Innovation Research commissioned by the Federal Ministry of Health summarized the health situation of persons with RD in Germany. This report highlighted the fact that these individuals continue to face significant limitations in healthcare provision despite major improvements such as the establishment of centers for rare diseases.^6^ Maternity care for women with RD was not included in this report. It may, however, be envisaged that pregnancy and childbirth put an additional challenge on the provision of adequate health care.

Women with pre‐existing medical conditions experience disproportionately higher rates of complications during pregnancy and childbirth which can be reduced through risk‐adapted care.^7^ This poses a particular challenge for women with RD, due to the lack of scientific evidence and clinical experience.

Literature on the experience of pregnancy in pre‐existing medical conditions is scarce but shows that women with chronic conditions experience becoming a mother as something unique and special but also face distinctive challenges such as concerns about their own health and that of their child.^8^, ^9^ Particularly for RD, the lack of experience among healthcare providers and midwives leads to additional conflicts related to pregnancy, childbirth, and postpartum care.^10^ Advances in medicine, including innovative medications and therapies, such as a newly approved CFTR modulator therapy for cystic fibrosis, offer improved prospects for affected women and increase the significance and relevance of family planning and pregnancies.^11^

A limited number of case series and reports on pregnancies in specific RD are available,^12^, ^13^, ^14^, ^15^, ^16^, ^17^ but data on maternity care and obstetric outcome in the overall group of RD do not exist.

Aim of this study was to evaluate maternal, obstetric, and perinatal outcomes of pregnancies in women with RD, recognizing that despite their heterogeneity these conditions share important pregnancy‐related features such as limited clinical experience, lack of robust guidelines, and frequent need for individualized multidisciplinary care.

## 2 MATERIAL AND METHODS

Retrospective analysis of pregnancies in women diagnosed with at least one RD who were cared for at our university hospital center with level IV of maternal care^18^ between January 2018 and August 2022. We applied the European definition of a RD (a condition that affects no more than 1 person per 2000 in the European population), as established by the European Union Regulation on Orphan Medicinal Products (1999).^19^ Cases were identified through the institutional database. Obstetric, perinatal and neonatal parameters were extracted from the Viewpoint database (Version 6, Munich, Germany) and data on maternal RD from the electronic patient record system (Orbis Version 32, Dedalus, Bonn, Germany).

Ethical review and approval were waived by the Ethics Committee at the Medical Faculty of Bonn (Ref.: 302/23‐EP) in view of the retrospective nature of the study. Informed consent was not obtained because of the study's retrospective design, and no patients were contacted during its conduct.

We recorded severe maternal morbidity (SMM) according to the criteria by the CDC,^20^ with the inclusion of transfusion of ≥4 blood products,^21^ and maternal, obstetric, perinatal, and neonatal parameters. Disease‐specific complication was defined as requiring inpatient or outpatient treatment for the underlying rare condition and preconception stability was defined as the absence of any disease‐specific complication in the 6 months preceding pregnancy.

Excessive blood loss was defined as >500 mL for vaginal birth and >1000 mL for Cesarean Section (CS).

Pregnancy‐specific complications included gestational diabetes (GDM), hypertensive disorders of pregnancy, intrahepatic cholestasis of pregnancy (ICP), gestational thrombocytopenia, and placenta previa.

Perinatal and neonatal outcomes were gestational week at birth, prematurity defined as gestational week <37 + 0, 5‐min Apgar score <7, neonatal intensive care unit (NICU) admission, congenital disorders and perinatal mortality.

Preconception counseling was defined as a documented consultation at our center prior to pregnancy with a maternal–fetal medicine specialist. Counseling was carried out in collaboration with specialists from the relevant disciplines.

Data on mode of conception was not available.

RD were categorized by organ system following Orphanet^19^ and also classified according to their etiology as follows: (A) Acquired, (B) Congenital excluding malformations, (C) Malformations, (D) Tumors.

Data analysis was performed using IBM SPSS Version 29 (SPSS Inc., Chicago, IL, USA). In addition to descriptive statistics, categorical subgroup comparisons were conducted using Pearson's chi‐squared test. When appropriate, exact tests were applied. Given the retrospective and descriptive nature of the study and the assessment of multiple maternal and perinatal outcomes, subgroup analyses were considered exploratory and *p*‐values were interpreted cautiously. A *p*‐value <0.05 was considered significant. Post‐hoc analysis using standardized residuals (SR) was performed with SR ≥2 considered indicative of over‐ or under‐representation.

In 12 patients, maternal and neonatal outcome data were missing.

## 3 RESULTS

**FIGURE 1:**
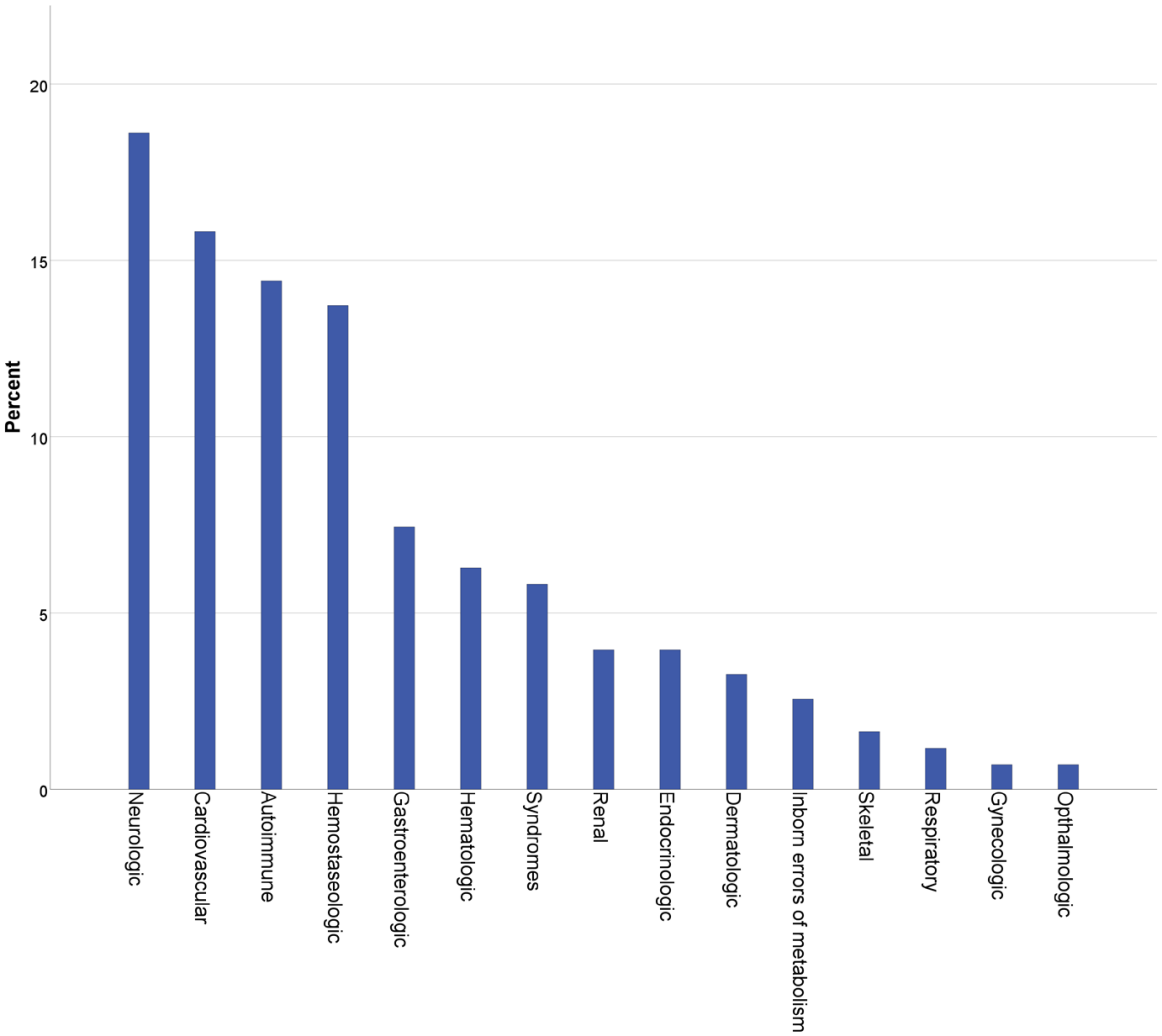
Rare Disease categorization according to organ system.

**FIGURE 2:**
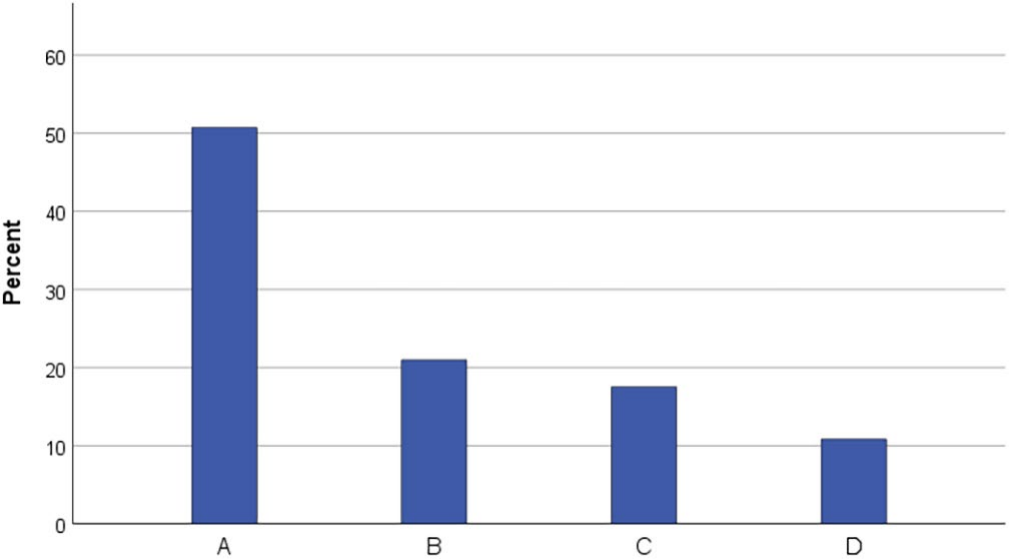
Rare Disease categorization according to etiology. A: Acquired; B: Congenital, excluding malformations; C: Malformations; D: Tumors.

During the observation period of 4.5 years, 388 pregnant women with RD were cared for. RD diagnoses (*n* = 434) are found in Table S1 and Figure 1. RD classified according to their etiology are found in Figure 2. In group D, 23.4% (*n* = 11) of patients had a history of benign tumors, whereas 76.6% (*n* = 36) had a history of malignant tumors. One patient received chemotherapy during pregnancy following a diagnosis of acute myeloid leukemia during gestation.

Patients' characteristics are listed in Table 1. 11.9% (*n* = 46) of women had more than one RD, and 46.1% (*n* = 179) had at least one additional non‐rare condition. In 5.7% (*n* = 22) of cases, initial diagnosis was established during pregnancy. Mean maternal age was 32.1 years (SD 5.2) and 98.2% had a singleton pregnancy.

**TABLE 1:** Overall patients' characteristics.

Variable	*N* (%) or median, (IQR)
Number of patients	388
Number of RD	434
Women with ≥2 rare diseases	46 (11.9)
On medication	156 (40.2)
Preconception stable disease	322 (85.2)
Maternal age (years)	
18–34	250 (64.4)
≥35	138 (35.6)
BMI pre‐pregnancy (kg/m^2^)	
<18.5	24 (6.2)
18.5–24.9	207 (53.3)
25–29.9	90 (23.2)
30–34.9	36 (9.3)
≥35	31 (8.0)
Parity	
0	215 (55.4)
1	113 (29.1)
≥2	60 (15.5)
Pregnancy‐specific complications	92 (25.1)
GDM	57 (15.6)
Hypertensive disorders of pregnancy	23 (6.3)
ICP	7 (1.9)
Placenta previa	6 (1.6)
Gestational thrombocytopenia	2 (0.6)
Delivery and maternal outcomes	
Disease‐specific complications during pregnancy	90 (23.2)
SMM	5 (1.3)
ICU admission	12 (3.1)
Mode of birth: CS	185 (50.6)
RD as indication for CS	29 (7.9)
Excessive blood loss	49 (12.6)
Perinatal and neonatal outcomes	
Gestational age (weeks; SD)	38.4 (±2.3)
Preterm birth (<37 + 0 weeks)	56 (15.3)
5‐min Apgar score <7	11 (3.0)
NICU admission	75 (20.4)
Congenital disorders	32 (8.7)
Postpartum outcomes	
Commenced breastfeeding	312 (88.9)
Median length of hospital stay after birth (days)	3 (1)
Pregnancy outcome	
Live birth	366 (94.3)
Pregnancy loss (gestational week <24)	4 (1.0)
Perinatal death^a^	5 (1.3)
Termination of pregnancy^b^	1 (0.3)
Missing	12 (3.1)

*Note*: Pregnancy‐specific complications and maternal, perinatal and neonatal outcomes in pregnancies of women with rare diseases (RD).

Abbreviations: CS, cesarean section; GDM, gestational diabetes mellitus; ICP, intrahepatic cholestasis of pregnancy; ICU, intensive care unit; NICU, neonatal intensive care unit.

^a^ Three intrauterine fetal deaths (24, 25, and 37 weeks of gestation and two early neonatal deaths) following delivery at gestational weeks 24 and 39.

^b^ Termination of pregnancy in gestational week 20 after diagnosis of the underlying disease during pregnancy.

5.9% (*n* = 23) of our patients had received preconception counseling at the study center, provided by a maternal–fetal medicine specialist. The time between preconception counseling and pregnancy was not evaluated.

Maternal outcomes in subgroups (organ systems and etiology) are presented in Tables 2 and 3. RD‐specific complications occurred in 23.2% (*n* = 90) of women, without difference in subgroups (Tables 2 and 3). The risk of disease‐specific complications was not affected by the number of RD (*p* = 0.75). Women with preconceptionally stable conditions experienced significantly fewer complications (15.5% vs. 45.7%; *p* = 0.001). SMM occurred in five cases (1.3%), including three cases of sickle‐cell crisis and two cases of transfusion‐only SMM due to obstetric complications. SMM was associated with maternal transfer to the intensive care unit in three cases and prematurity in all live births (*p* = 0.001). 18.0% (*n* = 70) of women required admission during pregnancy. Causes for admission were obstetric in 60%, related to RD in 34.3%, and to the non‐rare condition in 5.7% of patients.

**TABLE 2:** Selected maternal and perinatal outcomes in women with rare diseases (RD), stratified by the most prevalent organ systems (*n* = 434).

Selected maternal and perinatal outcomes	Neurologic *n* (%) (81;18.7)	Cardiovascular *n* (%) (68;15.7)	Autoimmune *n* (%) (63;14.5)	Hemostaseologic *n* (%) (59;13.6)	*p*‐value
RD‐specific complications	17 (21.0)	14 (20.6)	14 (22.2)	10 (16.9)	0.99
Pregnancy‐related complications	23 (28.4)	11 (16.7)	12 (19.0)	18 (30.5)	0.11
CS	44 (56.8)	31 (47.7)	25 (41.0)	27 (46.6)	0.08
RD as indication for CS	7 (8.8)	16 (24.6)	0 (0)	2 (3.5)	0.001
Excessive blood loss	7 (8.8)	10 (15.4)	10 (15.9)	10 (16.9)	0.82
Preterm birth	11 (14.1)	14 (21.9)	7 (11.9)	5 (8.6)	0.09

*Note*: *p*‐values represent global comparisons across all organ groups.

Abbreviation: CS, cesarean section.

**TABLE 3:** Selected maternal and perinatal outcomes in women with rare diseases (RD), stratified by etiology (A = acquired, B = congenital excluding malformations, C = malformations, D = tumors, *n* = 434).

Selected maternal and perinatal outcomes	Acquired RD (A) *n* (%) 220 (50.7)	Congenital RD excluding malformations (B) *n* (%) 91 (21.0)	Malformations (C) *n* (%) 76 (17.5)	Tumors (D) *n* (%) (47;10.8)	*p*‐value
RD‐specific complications	16 (21.1)	20 (22.0)	50 (22.7)	4 (8.5)	0.18
Pregnancy‐ related complications	18 (24.0)	21 (23.3)	52 (23.7)	12 (26.1)	0.99
CS	38 (52.1)	39 (45.3)	106 (49.3)	19 (43.2)	0.74
RD as indication for CS	12 (16.4)	9 (10.6)	14 (6.5)	1 (2.2)	0.02
Excessive blood loss	8 (11.0)	12 (13.6)	29 (13.2)	8 (17.8)	0.78
Preterm birth	15 (20.8)	11 (12.9)	33 (15.5)	5 (11.4)	0.46

Abbreviation: CS, cesarean section.

Pregnancy‐specific complications occurred in 25.1% of live births (*n* = 92) (Table 1) without difference in subgroups (Tables 2 and 3). GDM was observed in 15.6% of patients and did not differ across organ systems or etiological subgroups (*p* = 0.38 and *p* = 0.99, respectively). Hypertensive disorders of pregnancy occurred in 6.3% of cases (*n* = 23) and similarly showed no differences across organ systems or etiological subgroups (*p* = 0.33 and *p* = 0.74, respectively). ICP was observed in 1.9% of cases (*n* = 7); within the gastroenterological subgroup, 9.7% developed ICP. Rates did not differ across etiological subgroups (*p* = 0.56). Notably, 33.3% of ICP cases occurred in women with rare gastroenterological or hepatic diseases; however, this difference did not reach statistical significance across organ systems (*p* = 0.45).

Overall perinatal and neonatal outcomes are presented in Table 1. There were no differences in CS rates, excessive blood loss, or prematurity across subgroups (Tables 2 and 3). The proportion of neonates with an Apgar score <7 also did not differ between groups (*p* = 0.35 and *p* = 0.69). In 7.9% (*n* = 29) of cases, the RD was the indication for delivery by CS. Analysis by organ groups demonstrated a significant association between the RD as an indication for CS (Table 2; *p* = 0.001). This association was driven almost exclusively by the cardiovascular subgroup (SR = 4.4). We also observed an association between RD as the indication for CS and the etiological disease classification (Table 3; *p* = 0.02). This association was overrepresented in classification A (acquired disease) (SR = 2.3). Congenital disorders were diagnosed in 8.7% (*n* = 32) of newborns. 11.1% of patients decided not to breastfeed, nearly half of them (48.8%) due to the current medication.

## 4 DISCUSSION

In our retrospective analysis of pregnancy and childbirth in women with RD, we found a high live birth rate of 94.3%. Preterm birth (15.3%) and CS rates (50.6%) were increased. Preconception stability was associated with a significantly lower rate of disease‐specific complications.

Overall, approximately 15%–25% of confirmed pregnancies result in pregnancy loss.^22^ We observed a miscarriage rate of only 1.0%. We assume that the majority of early pregnancy losses are managed prior to the initial consultation at our department. Chromosomal abnormalities dominate the etiology of first‐trimester miscarriages. Further contributing immunological factors of certain RD such as systemic lupus erythematosus (SLE) and antiphospholipid syndrome are discussed in the literature.^23^

Half of the women in our study group delivered by CS, a considerably higher rate compared to the CS rate in Germany (31% in the year 2021).^24^ In 7.9% (*n* = 29) of our cases, the underlying RD led to the indication for CS. For several RD, a higher CS rate has been reported. Underlying causes may include a higher rate of obstetric complications (e.g., in neurofibromatosis^25^); cofactors like obesity (e.g., idiopathic intracranial hypertension^26^); and limiting factors of the RD itself (e.g., osteogenesis imperfecta^27^).

With 15.3% deliveries occurring prematurely, the preterm birth rate was also increased (for Germany in the year 2021: 7.9%^24^).

In the absence of population‐based outcome data for pregnancy in women with RD as a unified group, we also interpreted our findings in the context of studies addressing pre‐existing chronic diseases overall, as well as neurological, cardiovascular, and autoimmune diseases, which represent the largest disease groups in our cohort.

In a large population‐based study using the Netherlands Perinatal Registry, Rosman et al. reported a preterm birth rate of 9.2% among women with pre‐existing chronic medical conditions, compared with 4.9% in women without chronic disease, along with higher CS rates across all chronic disease categories (23.2%).^28^ In our cohort of pregnancies affected by RD, the preterm birth rate was higher at 15.3%, and more than half of women (50.6%) delivered by CS, exceeding rates reported in this population‐based study.^28^

Within the neurological disease subgroup, Rosman et al. included 3200 women and reported a CS rate of 17.3% and a preterm birth rate of 6.1%.^28^ In contrast, we observed higher rates of CS (56.8%; *n* = 44) and preterm birth (14.1%; *n* = 11) in our cohort.

In the Registry of Pregnancy and Cardiac Disease (ROPAC), the largest prospective international cohort of pregnant women with structural or ischemic heart disease, adverse obstetric and maternal outcomes were frequent, with preterm birth occurring in 13–36% of pregnancies, an overall CS rate of 41%, and maternal cardiac complications reported in 14% of cases in developed countries.^29^ In our cohort, the preterm birth rate (21.9%) and CS rate (47.7%) among women with rare cardiac diseases were comparable, whereas the frequency of maternal cardiac complications (20.6%) was higher. The elevated CS rate observed in our cohort and in the literature^29^ may partly relate to our observation that in 24.6% of cases the underlying cardiovascular disease itself was documented as the primary indication for CS.

Pregnancy outcomes in autoimmune diseases have been described in several studies.^30^ In our cohort of pregnancies affected by rare autoimmune diseases, including systemic lupus erythematosus (SLE) as the most prevalent diagnosis, disease‐specific complications occurred in 22.2% of cases, the CS rate was 41.0%, and preterm birth occurred in 11.9%. In a recent Austrian population‐based study, Rosta et al. compared pregnancy outcomes in women with SLE to both a high‐risk cohort and the general obstetric population, reporting higher rates of adverse outcomes in women with SLE than in the general population. This included maternal complications (28%), defined as a composite outcome encompassing pregnancy‐specific complications, postpartum complications, thromboembolic events, and other severe maternal outcomes, as well as CS (59%) and preterm birth (27%).^31^ Although the absolute rates observed in our cohort were lower, the overall pattern of increased maternal morbidity, obstetric intervention, and preterm birth is consistent with the elevated risks reported for SLE in this population‐based study.

Overall, these findings should be interpreted with caution, as differences may be influenced by variation in disease spectrum, severity, referral patterns, and the concentration of complex cases within our study population; however, they may also be related to a higher underlying disease burden among women with RD.

While disease‐specific analyses, such as those for cardiovascular RD—where a higher rate of CS is consistent with existing literature—can be directly interpreted in this context^29^ interpretation of findings across broader disease categories requires greater caution. The grouping of RD into acquired, congenital, malformations, and tumors was performed to enable structured analysis but does not represent clinically homogeneous entities. The reasons underlying the higher proportion of CS attributed to the RD in the acquired group remain unclear, and existing literature does not provide sufficient evidence to support a specific explanatory mechanism.

Pregnancy‐specific complications occurred in 25.1% of live births. GDM was observed in 15.6% of patients, which is comparable to the prevalence of 10.9% reported for the general population in Europe in a systematic review.^32^ We did not observe an increased rate of hypertensive disorders of pregnancy in the rare cardiovascular or autoimmune disease groups, in contrast to reports in the literature describing a higher risk in women with these pre‐existing conditions.^30^, ^33^ This discrepancy may be related to the limited number of cases within these subgroups. One‐third of cases of ICP occurred in women with pre‐existing gastroenterological or hepatic disease. An increased risk of ICP in the presence of underlying hepatobiliary disease has been reported in the literature.^34^

Research on pregnancies in multimorbid women (defined as having two or more chronic pre‐existing medical conditions) reveals a dose‐dependent relationship between the number of chronic conditions and the risk of adverse maternal outcome, including SMM and mortality.^7^, ^35^, ^36^ Whereas the average SMM rate is 1.3%, it increases to 5.6% in women with ≥3 pre‐existing conditions.^7^ The SMM rate in our patient cohort is in accordance with the literature. We could not confirm a relationship between the number of chronic conditions and SMM rate or disease‐specific complications, probably due to the size of our study group. SMM also adversely affects the perinatal outcome, increasing the likelihood of a 5‐min Apgar score <7, NICU admission, prematurity, and perinatal and neonatal mortality.^37^ In our study group, we observed a higher prematurity rate in women who suffered SMM (4/4 live births; *p* = 0.001).

The majority of women decided to breastfeed. This is in line with figures from Germany (87.3% in the year 2013).^38^ It is noteworthy that 45.5% of patients discontinued breastfeeding due to their current medication.

Only 5.9% of our patients had received preconception counseling at the study center. A systematic review by Nana et al. (2023) highlights the importance of preconception counseling for both the maternal and perinatal outcome.^39^ Additionally, in a study from the USA, Dude et al. (2022) found that routine examinations conducted in the year before conception for women with chronic conditions are associated with a reduced risk of maternal morbidity and mortality.^40^ In a review by Steel et al. (2015) of 14 studies, the proportion of women who received preconception counseling varied between 18.1% and 45%.^41^ Several guidelines^28^ recommend preconception counseling for specific conditions such as congenital heart disease, kidney disease, SLE, and antiphospholipid syndrome.^42^, ^43^, ^44^ Due to lack of experience and missing evidence from prospective randomized trials, especially patients with RD could benefit from structured interdisciplinary preconception counseling.

Preconception stability in pre‐existing medical conditions refers to the concept of aiming at an optimal state of chronic medical or psychological conditions before conception. Chronic conditions that are poorly controlled prior to conception can lead to disease‐specific and pregnancy‐associated complications. Although literature is limited, the stable phase of a disease before pregnancy appears to play an important role as predictor for a better pregnancy outcome in specific RD, among them SLE and autoimmune hepatitis.^45^, ^46^ We observed a significantly lower rate of disease‐specific complications if preconception stability was achieved, though numbers were small.

The rationale for pooling heterogeneous RD lies in the common denominators they share in the obstetric context: limited clinical evidence, complex care needs, and elevated risk of maternal and perinatal complications. While our analysis demonstrates that preconception stability is a strong predictor of improved outcomes, we acknowledge that grouping diverse conditions has inherent limitations. Disease‐specific risks may be obscured, and our results should therefore not be interpreted as interchangeable across all entities. Nonetheless, the pooled approach offers a valuable overview of the challenges women with RD face during pregnancy and highlights the need for both general obstetric and disease‐specific strategies. Reporting outcomes in the overall group of RD therefore provides a first step toward understanding the common challenges and informing healthcare planning.

The strengths of our study include the large cohort, the systematic categorization of RD by both organ system and etiology, and the detailed documentation of obstetric and perinatal outcomes. Limitations include the retrospective single‐center design, potential referral bias to a tertiary care center, and the heterogeneity of the study population, which limits disease‐specific conclusions. Furthermore, early pregnancy losses may be underrepresented, as these are often managed outside tertiary centers. These aspects must be taken into consideration when interpreting our findings.

## 5 CONCLUSION

In conclusion, the present study highlights the course and complications of pregnancies in patients with RD. The findings underscore the importance of preconception stability, as women with stable conditions prior to pregnancy experienced significantly fewer complications. Disease‐specific complications were common and did not vary by etiology or organ system. Likewise, pregnancy‐associated complications occurred in one‐quarter of cases.

These findings support the relevance of preconception disease optimization and coordinated multidisciplinary care in pregnancies complicated by rare diseases. Further prospective studies are needed to evaluate structured approaches in this population.

## AUTHORS CONTRIBUTIONS

Conceptualization: P.K., W.M., L.G., T.B., K.N., E.J. Methodology: P.K., W.M., E.J. Formal analysis: P.K. and K.N. Investigation: P.K., L.G., T.B., W.M., K.N. Data curation: P.K., W.M., and U.G. Writing—original draft preparation: P.K. and W.M. Writing—review and editing: P.K, K.N., L.G., T.B., U.G., W.M., E.J. Visualization: P.K., E.J. Supervision: W.M. All authors have read and agreed to the published version of the manuscript.

## FUNDING INFORMATION

This research received no external funding.

## CONFLICT OF INTEREST STATEMENT

The authors declare that they have no competing interests.

## ETHICS STATEMENT

Ethical review and approval were waived by the Ethics Committee at the Medical Faculty of Bonn (Ref.: 302/23‐EP) in view of the retrospective nature of the study.

## Supporting information


**Table S1.** Rare disease diagnoses (*n* = 434) in the study group of 388 women categorized by organ system and in individual subgroups according to frequency.

## Data Availability

The data that support the findings of this study are available from the corresponding author upon reasonable request.
