# Supplementary material for: Pregnancies in women with rare diseases: Selected maternal and perinatal outcomes
Source: Acta Obstet Gynecol Scand. 2026 Apr 8;105(6):1088–95. doi: 10.1111/aogs.70201 (PMC13191797; doi:10.1111/aogs.70201)
Supplement: Supplementary file 1 — Table S1. Rare disease diagnoses (n = 434) in the study group of 388 women categorized by organ system and in individual subgroups according to frequency. [file AOGS-105-1088-s001.docx]

| Neurologic  n=80 (18.6%) | Idiopathic intracranial hypertension (n=7), Cerebral sinovenous thrombosis (n=6), myasthenia gravis (n=6), Neurofibromatosis type 1 (n=6), Benign partial epilepsy of infancy with complex partial seizures (n=3), meningioma (n=3), Trigeminal neuralgia (n=3), Familial cerebral cavernous malformation (n=2), Ganglioglioma (n=2), Pediatric arterial ischemic stroke (n=2), Pediatric multiple sclerosis (n=2) Pilocytic astrocytoma (n=2), Segawa syndrome (n=2), Self-limited infantile epilepsy (n=2), Syringomyelia (n=2), Acoustic neurinoma (n=1), Benign partial epilepsy with secondarily generalized seizures in infancy (n=1), Bethlem muscular dystrophy (n=1), Cerebral arteriovenous malformation (n=1), Childhood absence epilepsy (n=1), Chronic inflammatory demyelinating polyneuropathy (n=1), Cogan syndrome (n=1), Closed spinal dysraphism (n=1), Congenital cerebral angioma (n=1), Cramp-fasciculation syndrome (n=1), Dopa-responsive dystonia (n=1), Duchenne muscular dystrophy (n=1), Familial temporal lobe epilepsy (n=1), Hereditary hyperekplexia (n=1), Herpes simplex virus encephalitis (n=1), Isolated Dandy-Walker malformation with hydrocephalus (n=1), Mega-cisterna magna (n=1), Mesial temporal lobe epilepsy with hippocampal sclerosis (n=1), Myotonia congenita (n=1), Narcolepsy (n=1), Oligoastrocytoma (n=1), Open spinal dysraphism (n=1) Parsonage-Turner Syndrome (n=1), Pituitary dermoid and epidermoid cysts (n=1), Poliomyelitis (n=1), Proximal myotonic myopathy (n=1), Proximal spinal muscular atrophy type 3 (n=1), Pudendal nerve entrapment syndrome (n=1), Rasmussen subacute encephalitis (n=1) |
| --- | --- |
| Cardiovascular  n=68 (15.8%) | Atrial septal defect (n=12), Coarctation of the aorta (n=9), Dextro-transposition of the great arteries (n=5), Congenital aortic valve stenosis (n=4), Long QT syndrome (n=4), Anomalous pulmonary venous connection (n=3), Pulmonary valve stenosis (n=3), Atrial septal aneurysm (n=2), Atrioventricular septal defect (n=2), Tricuspid atresia (n=2), Dilatative cardiomyopathy (n=2), Peripartum cardiomyopathy (n=2), Pulmonary arterial hypertension (n=2), Valvulopathy after rheumatic fever (n=2), Bland White Garland syndrome (n=1), Double outlet right ventricle (n=1), Ebstein anomaly (n=1), Hypertrophic cardiomyopathy (n=1), Left ventricular aneurysm (n=1), Infective endocarditis (n=1), Pulmonary atresia with ventricular septal defect (n=1), Scimitar syndrome (n=1), Subaortic stenosis (n=1), Tetralogy of Fallot (n=1), Sick sinus syndrome (n=1), Cardiac sarcoidosis (n=1), Coeliac artery compression syndrome (n=1), M. Osler (n=1) |
| Autoimmune  n=62 (14.4%) | Systemic lupus erythematosus (n=16), Sarcoidosis (n=10), Mixed connective tissue disease (n=5), Undifferentiated connective tissue syndrome (n=5), Indolent systemic mastocytosis (n=4), Sjögren's disease (n=4), Juvenile idiopathic arthritis (n=3), Sharp syndrome (n=2), Behçet's disease (n=2), Familial Mediterranean fever (n=2), Autoinflammatory syndrome (n=1) granulomatous mastitis (n=1), Henoch-Schönlein purpura (n=1), Hypocomplementemic urticarial vasculitis (n=1), Idiopathic arthritis (n=1), Localized scleroderma (n=1), Monoclonal mast cell activation syndrome (n=1), Systemic sclerosis (n=1), Systemic-onset juvenile idiopathic arthritis (n=1) |
| Hemostaseologic  n=60 (13.9%) | Von Willebrand disease type 1 (n=22)**,** Immunthrombocytopenia (n=19), Factor VII deficiency (n=8), Female carriers of hemophilia (n=4), Essential thrombocytosis (n=1), Factor XII deficiency (n=1), Factor XIII deficiency (n=1), Gray platlet syndrome (n=1), Hereditary dysfibrinogenemia (n=1), Hypofibrinogenemia (n=1), Splenic vein thrombosis (n=1) |
| Gastroenterologic  n=31(7.2%) | Autoimmune hepatitis (n=7), Primary sclerosing cholangitis (n=3), Non-malignant and non-cirrhotic portal vein thrombosis (n=3), Budd-Chiari syndrome (n=2), Attenuated familial adenomatous polyposis (n=2), Hirschsprung disease (n=2), Primary biliary cholangitis (n=2), Adenocarcinoma of the small intestine (n=1), Classic neuroendocrine tumor of appendix (n=1), Distomatosis (n=1), Gastrointestinal stromal tumor (n=1), Hepatocellular carcinoma (n=1), Non-functioning neuroendocrine tumor of pancreas (n=1), Proton-pump inhibitor-responsive esophageal eosinophilia (n=1), Secondary short bowel syndrome (n=1), Serrated polyposis syndrome (n=1), Small bowel atresia (n=1) |
| Hematologic  n=26 (6.0%) | Hodgkin's lymphoma (n=6), Sickle cell disease (n=3), Acute lymphatic leukemia (n=3), Myeloproliferative disorder (n=2), Spherocytosis (n=2), Alpha thalassemia (n=1), Beta thalassemia (n=1), Acute myeloid leukemia (n=1), Burkitt Lymphom (n=1), Non-Hodgkin lymphoma (n=1), May-Hegglin anomaly (n=1), Chronic idiopathic neutropenia (n=1), Chronic myelogenous leukemia (n=1), Cyclic neutropenia (n=1), Polycythemia vera (n=1), |
| Syndromes  n=23 (5.4%) | Ehlers Danlos syndrome (hypermobile) (n=4), Heterotaxia (n=2), Von Hippel-Lindau disease (n=2), Marfan syndrome (n=2), Alagille syndrome (n=1), Alport Syndrome (n=1), Carney complex (n=1), Cat-Eye-Syndrome (n=1), Ehlers Danlos Syndrome (vascular) (n=1), Ehlers Danlos syndrome (classical) (n=1), Evans syndrome (n=1), Klippel-Trénaunay syndrome (n=1), Lynch syndrome (n=1), Peutz-Jeghers syndrome (n=1), Silver-Russell syndrome (n=1), Tubulointerstitial nephritis and uveitis syndrome (n=1), Situs inversus totalis (n=1) |
| Endocrinologic  n=18 (4.2%) | Differentiated thyroid carcinoma (n=9), Addison disease (n=4), Arginine vasopressin deficiency (n=2), Autoimmune polyendocrinopathy type 2 (n=1), Maturity-onset diabetes of the young Type II (n=1), Multiple endocrine neoplasia type 1 (n=1) |
| Renal  n=17 (4.0%) | Autosomal dominant polycystic kidney disease (n=6), Interstitial cystitis (n=2), Renal or urinary tract malformation (n=2), Atypical HUS (n=1), Goodpasture syndrome (n=1), Distal renal tubular acidosis (n=1) Immunoglobulin-mediated membranoproliferative glomerulonephritis (n=1), Primary membranous glomerulonephritis (n=1), Renal hypoplasia (n=1), Renal nutcracker syndrome (n=1) |
| Dermatologic  n=15 (3.5%) | Rare cutaneous lupus erythematosus (n=6), Hereditary angioedema (n=4), Localized scleroderma (n=2), Chilblain lupus (n=1), Dystrophic epidermolysis bullosa (n=1), Nodular non-suppurative panniculitis (n=1) |
| Inborn errors of metabolism  n=12 (2.8%) | Congenital adrenal hyperplasia (n=4), Phenylketonuria (n=2), Acute intermittent porphyria (n=1), Alpha-1-antitrypsin deficiency (n=1), Cystic fibrosis (n=1), Glycogen storage disease type Ib (n=1), Multiple acyl-CoA dehydrogenase deficiency (n=1), X-linked hypophosphatemia (n=1) |
| Skeletal  n=7 (1.6%) | Skeletal Ewing sarcoma (n=2), Achondroplasia (n=1), Giant cell tumor of bone (n=1), Osteogenesis imperfecta type 3 (n=1), Osteosarcoma (n=1), Split cord malformation (n=1) |
| Respiratory  n=5 (1.2%) | Tuberculosis (n=3), Idiopathic eosinophilic pneumonia (n=1), Pleural empyema (n=1) |
| Opthalmologic  n=3 (0.7%) | Central retinal vein occlusion (n=1), Herpes simplex virus stromal keratitis (n=1), Retinoblastoma (n=1) |
| Gynecologic  n=3 (0.7%) | Unicervical bicornuate uterus (n=3) |

**Table S1**: Rare disease diagnoses (n=434) in the study group of 388 women categorized by organ system and in individual subgroups according to frequency
